# *MaMYBR30*, a Novel 1R-MYB, Plays Important Roles in Plant Development and Abiotic Stress Resistance

**DOI:** 10.3390/plants13131794

**Published:** 2024-06-28

**Authors:** Li Liu, Shan Li, Fengjuan Tang, Peijun Li, Jiaxin Liu, Rumeng Fu, Longyan Zheng, Jie Zhang, Nan Chao

**Affiliations:** 1 Jiangsu Key Laboratory of Sericultural Biology and Biotechnology, School of Biotechnology, Jiangsu University of Science and Technology, Zhenjiang 212100, China221211801106@stu.just.edu.cn (F.T.); 221211803106@stu.just.edu.cn (J.L.); 221211802132@just.edu.cn (J.Z.); 2 Key Laboratory of Silkworm and Mulberry Genetic Improvement, Ministry of Agriculture and Rural Affairs, Sericultural Research Institute, Chinese Academy of Agricultural Sciences, Zhenjiang 212100, China

**Keywords:** drought stress, co-expression, ion toxicity, MYB transcription factor, salt stress

## Abstract

The V-myb myeloblastosis viral oncogene homolog (MYB) family participate in various bioprocesses including development and abiotic stress responses. In the present study, we first report a 1R SHAQKYF-class MYB, *MaMYBR30*, in mulberry. Subcellular localization and sequence analysis indicated *MaMYBR30* is located in the nucleus and belongs to a CCA-like subgroup with a conserved SHAQKYF motif. Expression profile analysis showed that *MaMYBR30* is expressed in leaves and can be induced by drought and salt stress. The down-regulation of *MaMYBR30* using virus-induced gene silence (VIGS) in mulberry and the overexpression of *MaMYBR30* in *Arabidopsis* were induced to explore the function of *MaMYBR30*. The functional characterization of *MaMYBR30* in vivo indicated that *MaMYBR30* can positively regulate the resistance of mulberry to drought while negatively regulating the resistance of mulberry to salt stress. In addition, *MaMYBR30* also affects flower development and reproductive growth, especially after exposure to salt stress. Weighted gene co-expression network analysis (WGCNA) primarily revealed the possible genes and signal pathways that are regulated by *MaMYBR30*. Our results also imply that complex molecular mechanisms mediated by *MaMYBR30*, including crosstalk of ion toxicity, phytohormone signal transduction, flowering development, and epigenetic modification, need to be further explored in the future.

## 1. Introduction

The V-myb myeloblastosis viral oncogene homolog (MYB) family is one of the largest families of transcriptional regulators in plants and has been widely characterized [1]. The important roles of MYBs in plant development, abiotic and biotic stress resistance, and secondary metabolism have been characterized in diverse plants [1,2,3]. MYB proteins contain a highly conserved DNA-binding domain in the N-terminus with different numbers of repeats that contain regularly spaced tryptophan residues (WX18–19WX18–19W) [1,4]. The MYBs identified in plants are divided into four clades based on the number of conserved DNA-binding repeats: 1R (R1/2, R3-MYB), 2R (R2R3-MYB), 3R (R1R2R3-MYB), and 4R -MYB [2,5]. The 3R-MYBs and 4R-MYBs are relatively rare, and most MYBs identified and functionally characterized are R2R3-MYBs and partial 1R-MYBs [2,5].

The 1R-MYBs (R3-MYB), also called MYB-related genes, comprise another diverse group of MYB transcription factors [1,6]. These MYBs contain only a single- or partial-repeat MYB DNA-binding domain and can be further divided into five distinct subgroups depending on their conserved motifs [7]. The TRF-like and TBP-like subgroup MYBs always contain three conserved Trp (W) residues in the MYB domain. In CCA1-like/R-R and I-box-like subgroups, the third W residue is often substituted by Ala (A) or Tyr (Y), respectively [7]. In most members of the CPC-like subgroup, the first W residue is substituted with Phe (F). MYBs in the CCA1-like/R-R subgroup have highly conserved consensus sequences of SHAQK(+Y/F)F in their MYB domains and are also called SHAQKYF-class MYBs [7]. In rice, SHALLOT-LIKE1 (SLL1) encodes a SHAQKYF-class MYB family transcription factor belonging to the KANADI family and regulates leaf abaxial cell development to shape the leaf and control rice leaf rolling [8]. SHAQKYF-class MYBs can also act as transcription repressors that regulate leaf wax biosynthesis via transcriptional suppression on DEWAX in *Arabidopsis* [9]. Some 1R-MYBs in maize have also shown possible roles in responses to drought stress and pathogen infection [7]. CCA1-like/R-R genes (*ZmMYBR19*, *ZmMYBR28*, *ZmMYBR49*, and *ZmMYBR56*), TRF-like genes (*ZmMYBR41*), and TBP-like genes (*ZmMYBR07*, *ZmMYBR26*, *ZmMYBR31*, *ZmMYBR45*, *ZmMYBR47*, and *ZmMYBR55*) have shown enhanced transcription levels under drought stress [7]. In general, 1R-MYBs also play important roles in diverse bioprocesses, and more studies are needed to reveal their function in plants.

Mulberry (*Morus alba* L.) is a traditional economic tree in China and shows potential value in ecological improvement, especially in drought- or salt-affected areas [10,11,12,13,14]. Here, we first report *MaMYBR30*, a 1R-SHAQKYF-class MYB, that greatly affects the resistance of mulberry to drought and high salt stress. Our previous study identified the MYB gene family in *Morus* and provided primary functional annotation of most MYBs [15]. Based on this study, we further screened MYBs involved in the drought stress response in *M. alba,* and *MaMYBR30* was identified based on weighted gene co-expression network analysis (WGCNA). Further functional characterization of *MaMYBR30* in vivo indicated that *MaMYBR30* can regulate the resistance of mulberry to drought and salt stress and can also affect the reproductive process.

## 2. Results

### 2.1. Comparative RNA-Seq Analysis Indicates That MaMYBR30 Is Involved in Drought Stress Responses

**Figure 1:**
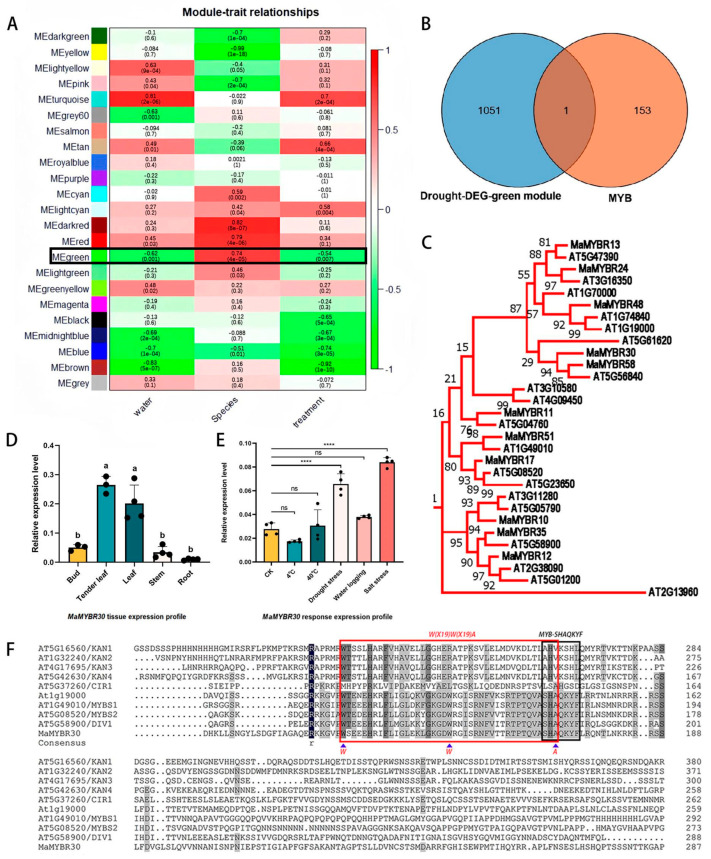
CCA1-like clade *MaMYBR30* is involved in drought and salt stress responses. (**A**). WGCNA indicates the modules’ and the traits’ relationships; species indicate Zhongshen 1 and Wubu varieties and treatment indicates the drought treatments. (**B**). Venn diagram of DEGs in MEgreen module and the MYBs in mulberry. (**C**). Phylogenetic analysis of *MaMYBR30* and CCA1-like MYB from *Arabidopsis thaliana*. (**D**). Expression profile of *MaMYBR30* in different organs in mulberry. (**E**). Expression levels of *MaMYBR30* in response to different stresses. (**F**). Alignment of *MaMYBR30* and 1-R MYB from *Arabidopsis thaliana*. Data are presented as means ± SD of at least three biological replicates. The significance was marked using **** (*p* < 0.0001) or indicated by different letters.

The comparative transcriptome analysis of mulberry cultivars (Zhongshen 1 and Wubu) with different levels of drought tolerance identified drought-related differentially expressed genes (DEGs). The difference in drought tolerance between Zhongshen 1 and Wubu was revealed in our previous study, and the expression matrix is shown in Table S1 [16]. The DEGs contained 44 MYBs annotated as abiotic stress-related MYBs in our previous study in mulberry (Figure S1 and Table S2) [15]. Furthermore, using WGCNA, drought-related DEGs were clustered into 23 co-expression modules. Among them, the MEgreen module showed a significant positive relationship with species and a negative relationship with water content and treatment (Figure 1A). Therefore, we identified this module as a cultivar-specific positive drought-response module. Venn analysis of DEGs in the MEgreen module and MYBs in *Morus* revealed only one MYB-related gene, *MaMYBR30*, indicating its possible roles in the response to drought stress and the difference in drought tolerance between Zhonsghen1 and Wubu (Figure 1B).

### 2.2. Mulberry MaMYBR30 Belongs to SHAQKY-Class MYBs and Is Involved in Drought and Salt Stress Responses

**Figure 2:**
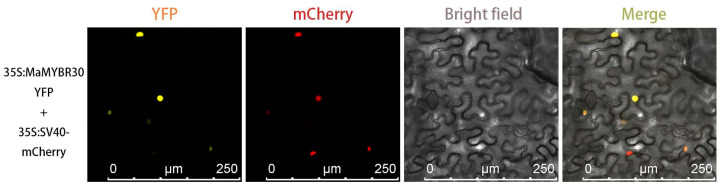
Subcellular localization of *MYBR30*. The nucleus location marker SV40-mCherry signals merged with *MaMYBR30*-YFP signals.

*MaMYBR30* was cloned from the *M. alba* variety *Fengchi*. Sequence alignment and phylogenetic analysis showed that *MaMYBR30* was a SHAQKY-class MYB belonging to the CCA1-like subgroup. The substitution of the third W by A in the MYB domain is marked, and a conserved SHAQKY motif is also indicated (Figure 1F). Phylogenetic analysis using 1R-MaMYB from mulberry and 1R-AtMYB from *Arabidopsis* showed that *MaMYBR30* clustered with CCA1-like subgroup AtMYBs (Figure 1C and Figure S2). Expression profile analysis showed that *MaMYBR30* was expressed in leaves with significantly increased expression levels under drought or high salt stress (Figure 1D,E). In addition, subcellular localization analysis was performed and showed that the YFP signals of *MYBR30*-YFP merged with the nucleus marker signal (Figure 2). This result indicates the nucleus localization of *MYBR30*, which corresponds with its role as a transcription factor.

### 2.3. MaMYBR30 Down-Regulation Decreases Drought Stress Resistance in Mulberry

**Figure 3:**
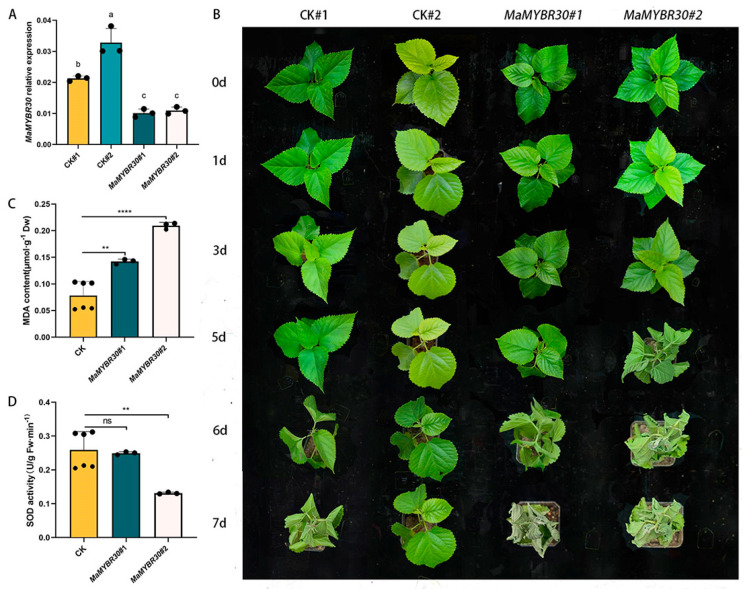
Down-regulation of *MaMYBR30* by VIGS decreases drought tolerance in mulberry. (**A**). qRT-PCR detection of *MaMYBR30* expression levels in VIGS-treated mulberry plants. (**B**). Growth conditions of VIGS-treated mulberry and controls under drought stress; plants were observed and recorded until the seventh day after exposure to drought stress. (**C**). MDA contents in VIGS-treated mulberry and controls after exposure to drought stress. (**D**). SOD activities in VIGS-treated mulberry and controls after exposure to drought stress. MDA and SOD were determined in leaves on the seventh day of stress treatment. CK: mulberry plants treated with empty vectors were used as controls; CK#1 and 2 indicated two independent CKs as biological replicates. MaMYBR30 #1 and 2: independent mulberry plants with the down-regulation of MaMYBR30 by VIGS treatment. Data are presented as means ± SD of three biological replicates. The significance was marked using ** (0.001 < *p* < 0.01), and **** (*p* < 0.0001) or indicated by different letters.

qRT-PCR was performed to test the degree of down-regulation after VIGS (Figure 3A). On day 5 after exposure to drought stress, mulberry seedlings showing the down-regulation of *MaMYBR30* had wilted and coiled leaves, while the controls remained normal. On day 7, seedlings with *MaMYBR30* down-regulation were almost dead, while the controls showed only slight wilting (Figure 3B). Mulberry with *MaMYBR30* down-regulation showed significantly higher MDA content and similar SOD activity compared with the controls (Figure 3C,D).

### 2.4. MaMYBR30 Down-Regulation Increases Salt Stress Resistance in Mulberry

**Figure 4:**
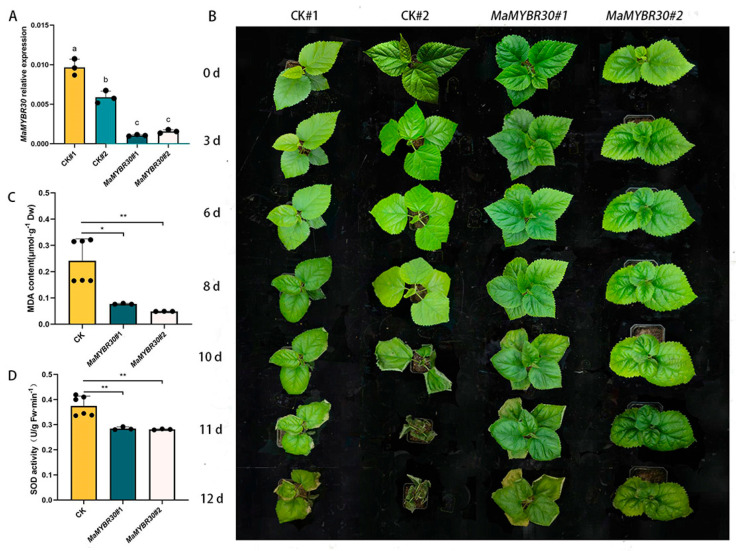
*MaMYBR30* down-regulation by VIGS decreases tolerance to high salt stress. (**A**). qRT−PCR detection of *MaMYBR30* expression levels in VIGS-treated mulberry plants. (**B**). Growth conditions of VIGS−treated mulberry and controls under high salt stress; plants were observed and recorded until the twelfth day after exposure to drought stress. (**C**). MDA content in VIGS-treated mulberry and controls after exposure to high salt stress. (**D**). SOD activity in VIGS−treated mulberry and controls after exposure to high salt stress. MDA and SOD were determined in leaves on the twelfth day of stress treatment. CK: mulberry plants treated with empty vectors were used as controls; CK#1 and 2 indicated two independent CKs as biological replicates. MaMYBR30 #1 and 2: independent mulberry plants with the down-regulation of *MaMYBR30* by VIGS treatment. Data are presented as means ± SD of three biological replicates. Significant differences are marked using * (0.01 < *p* < 0.05), and ** (0.001 < *p* < 0.01) or indicated by different letters.

qRT-PCR was performed to test the degree of down-regulation after VIGS (Figure 4A). Mulberry seedlings with *MaMYBR30* down-regulation and controls were exposed to high salt stress. On day 10, the leaves of the controls gradually withered and coiled (Figure 4A,B). This phenome was further aggravated over the following two days and resulted in plant death. In contrast, the leaves of mulberry seedlings with *MaMYBR30* down-regulation became slightly wilted and turned dark green (Figure 4B); they showed significantly lower MDA content and SOD activity compared with the controls (Figure 4C,D). *MaMYBR30* down-regulation likely increased resistance to high salt stress in mulberry, which is quite different from its role in response to drought stress.

### 2.5. MaMYBR30 Overexpression in Arabidopsis Indicates Its Contrasting Roles in Regulating Drought and Salt Stress Resistance

**Figure 5:**
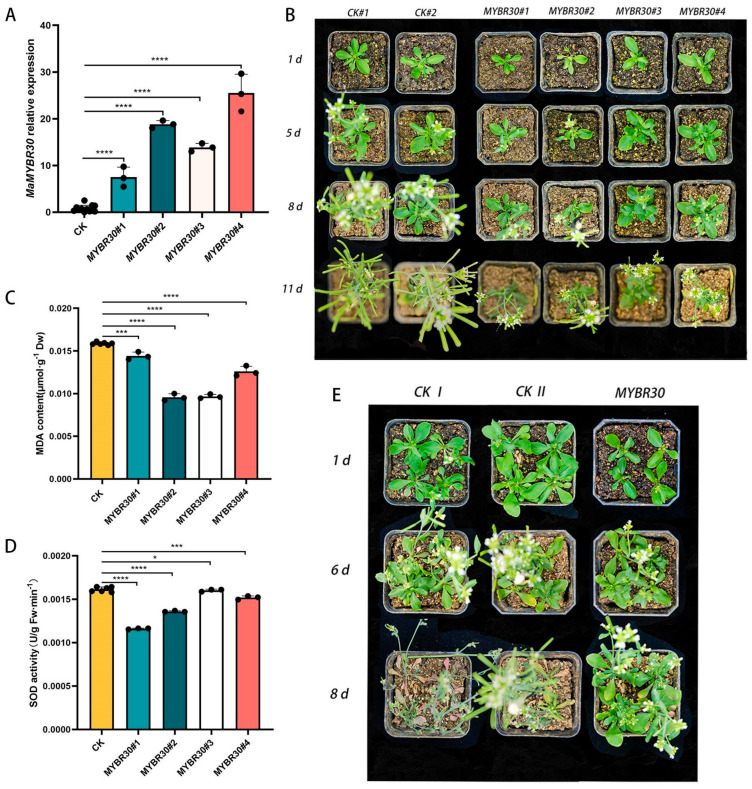
*MaMYBR30*-overexpressing *Arabidopsis* enhanced plant drought tolerance. (**A**). qRT−PCR detection of *MaMYBR30* expression levels in transgenic *Arabidopsis* lines. (**B**). Growth conditions of transgenic *Arabidopsis* (one line in one pot) and wild types under drought stress for indicated period; (**C**). MDA contents in transgenic *Arabidopsis* and wild types after exposure to drought stress. (**D**). SOD activities in transgenic *Arabidopsis* and wild types after exposure to drought stress. (**E**). Growth conditions of transgenic *Arabidopsis* (four lines in one pot) and wild types under drought stress. Wild types were used as the CK group. MDA and SOD were determined in leaves on the eighth day of stress treatment. Data are presented as means ± SD of three biological replicates. The significance was marked using * (0.01 < *p* < 0.05), *** (0.0001 < *p* < 0.001), and **** (*p* < 0.0001).

qRT-PCR was performed to test and validate the overexpression level of transgenic *Arabidopsis* plants (Figure 5A). *MaMYBR30*-overexpressing *Arabidopsis* maintained similar growth to that of the wild type (Figure S3A). Two kinds of planting strategies were adopted to observe the plant phenomics under drought stresses: both wild-type and over-expressing lines were planted by one pot with one plant (one line in one pot); four wild types or four plants from different overexpressing lines were planted by one with four plants (four lines in one pot). When exposed to drought stress, *MaMYBR30*-overexpressing *Arabidopsis* showed enhanced drought stress tolerance and remained alive after 11 days of drought stress, while the wild type showed significant growth inhibition (one line in one pot) (Figure 5B). Four lines planted in one pot group showed more obvious differences. Four *MaMYBR30*-overexpressing lines remained alive, while the wild type almost died after exposure to 8 days of drought stress (Figure 5E). Both the MDA content and SOD activity in *MaMYBR30*-overexpressing *Arabidopsis* were lower than in the wild type after 8 days of drought stress treatment (Figure 5C,D).

**Figure 6:**
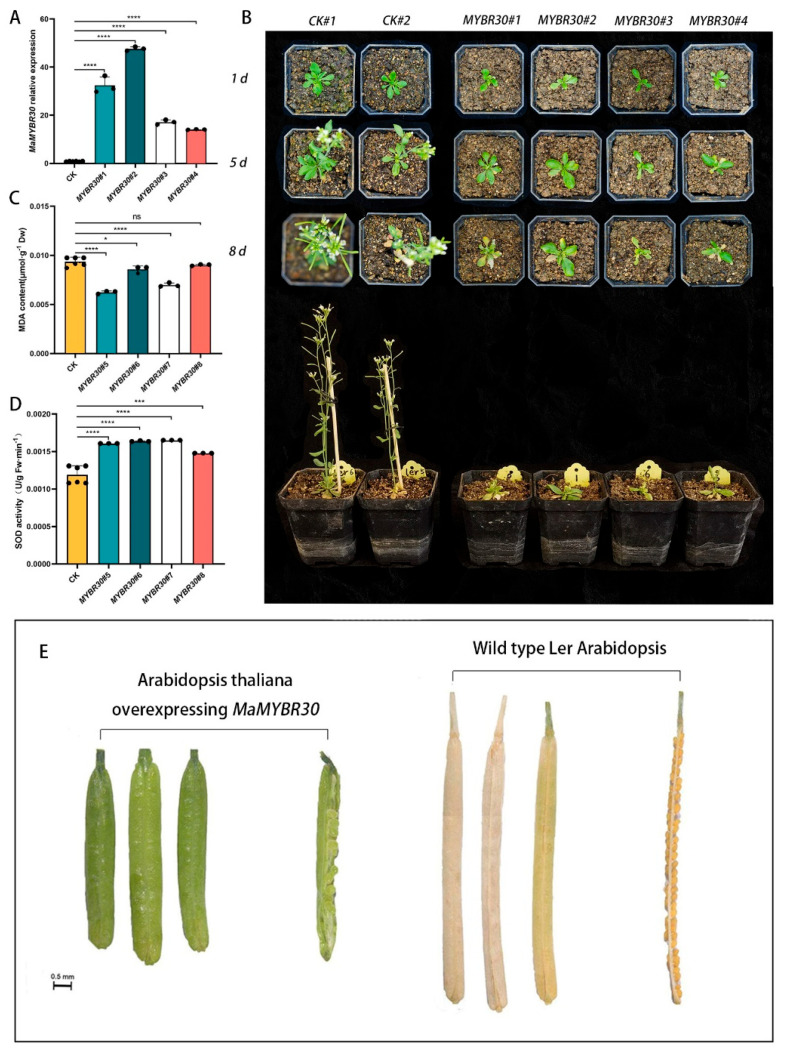
*MaMYBR30*-overexpressing *Arabidopsis* decreased plant salt tolerance. (**A**). qRT-PCR detection of *MaMYBR30* expression levels in transgenic and wild-type (CK) *Arabidopsis* lines. (**B**). Growth conditions of transgenic *Arabidopsis* (one line in one pot) and wild types under high salt stress for indicated periods. (**C**). MDA contents in transgenic *Arabidopsis* and wild types after exposure to high salt stress. (**D**). SOD activities in transgenic *Arabidopsis* and wild types after exposure to high salt stress. (**E**). Pods and seeds of transgenic *Arabidopsis* and wild types after recovery. The plants were irrigated with 150 mM NaCl instead of water. MDA and SOD were determined in leaves on the eighth day of stress treatment. Data are presented as means ± SD of three biological replicates. The significance was marked using * (0.01 < *p* < 0.05), *** (0.0001 < *p* < 0.001), and **** (*p* < 0.0001) or indicated by different letters.

Visible growth differences were observed between the wild-type and transgenic plants when supplied with 150–300 mM NaCl instead of water (Figure 6B and Figure S3C). The growth of *MaMYBR30*-overexpressing *Arabidopsis* (Figure 6A) was severely inhibited, with abnormal flowering and reproductive processes, under salt stress (150 mM NaCl; Figure 6B). In contrast, the wild type showed relatively normal flowering and reproductive growth. After recovery with water supply, the wild type almost recovered normal reproductive growth with normal pod and seed filling, while *MaMYBR30*-overexpressing *Arabidopsis* had stunted pods and few seeds with delays in seed ripening (Figure 6E and Figure S3D). *MaMYBR30* overexpression resulted in sensitivity to salt stress and the abnormal development of the reproductive process. The MDA content in *MYBR30*-overexpressing *Arabidopsis* plants was lower than that of the wild type, and the SOD activity was higher than that of the wild type (Figure 6C,D). This implies that the salinity sensitivity of *MaMYBR30*-overexpressing plants may be due to the priming effects of a high salt concentration, such as anion toxicity, instead of the indirect damage caused by osmotic stress.

### 2.6. Co-Expression Analysis Shows the Pathways Regulated by MYBR30

**Figure 7:**
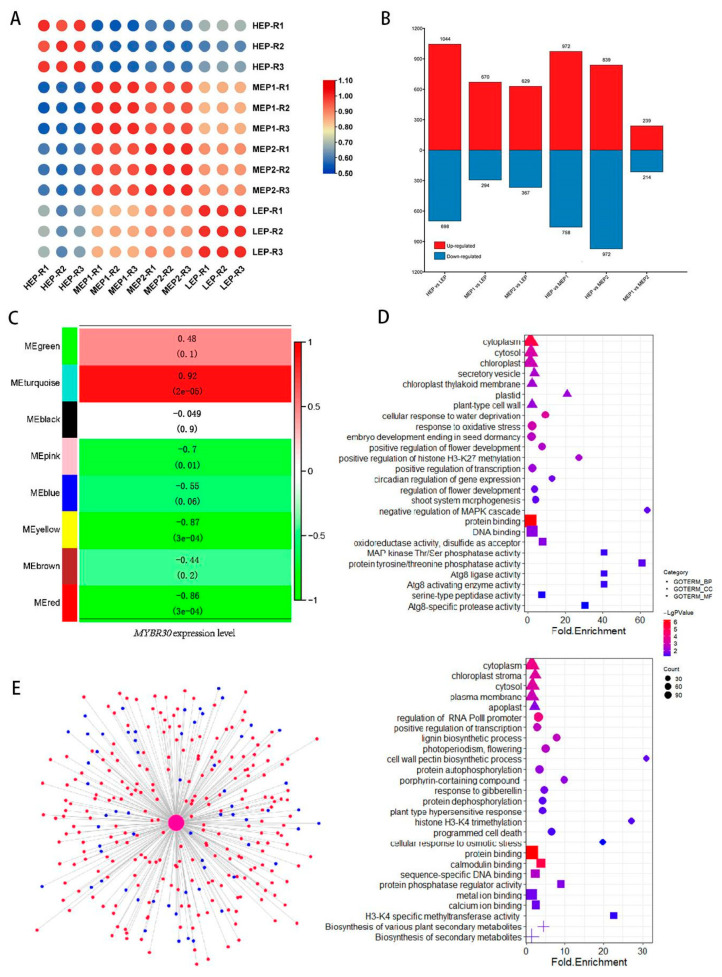
Comparative transcriptome analysis of mulberry plants with different *MaMYBR30* expression levels. (**A**). Correlation of RNA-seq data from mulberry plants with different *MaMYBR30* expression levels. HEP: high expression level of *MYBR30*; MEP: medium expression level of *MYBR30*; LEP: low expression level of *MYBR30*; (**B**). Number of up-regulated and down-regulated genes in different comparative groups. (**C**). Correlation of modules and traits. (**D**). GO and KEGG enrichment of DEGs in the MEturquoise module (up) and DEGs in MEred and MEyellow modules (bottom). “+” indicated KEGG pathways. (**E**). Co-expression network of *MaMYBR30* and top 300 related DEGs. The red circles indicate DEGs in the MEturquoise module, and the blue circles indicate DEGs in the MEred and MEyellow modules.

The change in MaMYBR30 expression level combined with co-expression analysis indicates the possible pathways mediated by *MaMYBR30*. Mulberry plants with different *MaMYBR30* expression levels were obtained using VIGS treatments. Four groups of mulberry plants with different *MaMYBR30* expression levels were collected: the high-expression group (HEP), medium-expression group 1 (MEP1), medium-expression group 2 (MEP2), and low-expression group (LEP) (Figure S4). Correlation analysis of all samples based on RNA-seq data showed that the different groups were well distinguished, and the samples in the same group showed high correlation (Figure 7A). DEGs in different comparative groups were identified, and the number of DEGs in HEP vs. LEP and HEP vs. MEP1/MEP2 was higher than in other comparative groups, indicating that the difference in *MYBR30* expression levels dominantly affects the DEG numbers (Figure 7B). WGCNA using all DEGs and the expression level of *MYBR30* as an associated trait showed that eight modules were clustered. Among these modules, MEturquoise showed a significant positive correlation (r = 0.92, *p* = 2 × E^−5^) with *MYBR30* and MEred (r = −0.85, *p* = 3 × E^−4^) and MEyellow (r = −0.87, *p* = 3 × E^−4^) showed a significant negative correlation with *MYBR30* (Figure 7C). GO and KEGG enrichment analysis using genes in MEturquoise or MEred and MEyellow, respectively, was performed. Genes in MEturquoise were enriched in bioprocesses involved in response to water deprivation and oxidation stress, indicating their roles in the response to drought stress (Figure 7D). Genes in MEred and MEyellow were involved in lignin biosynthesis, cell wall pectin biosynthesis, gibberellin response, and the calcium signal (Figure 7D). The calcium signal pathway plays an important role in mediating the signaling of various ionic stressors, and plants use calcium to resolve salt stress [17,18]. These differences show the possible different roles that *MYBR30* plays in response to drought or salt stress. In addition, both positive and negative related modules showed gene enrichment in bioprocesses, including flower, seed development, and epigenetic modification involved in flowering or vernalization. The MAPK signal pathway, which is responsible for osmotic stress signaling and initializing cellular processes, such as proliferation, differentiation, and development, was also enriched. Genes annotated as negative regulators of the MAPK cascade were also positively related to *MYBR30*. The MAPK signal pathway and epigenetic modification of flowering-related genes regulated by *MYBR30* may be mechanisms for the abnormal development of *MYBR30*-overexpressing *Arabidopsis* after exposure to salt stress. 

In addition, the top 300 *MYBR30*-related genes were selected from networks, and the genes involved in the calcium signal, stress responsiveness, flower development, and epigenetic modification were identified (Figure 7E and Figure S5; Table 1). For example, M.alba_G0001459 is a mulberry homolog gene of AtCDPK2 (CALCIUM-DEPENDENT PROTEIN KINASE 2). CDPK2 is known to play an important role in the calcium signal pathway, and its mutant in *Arabidopsis* has increased sensitivity to ABA and salt. M.alba_G0004262, which is a mulberry homolog gene of ATX1, is known for its activation of histone H3K4Me3 and Flowering Locus C (FLC). These star genes showed close co-expression with *MYBR30*, and further study should be carried out to reveal the mechanism of signal pathways mediated by *MYBR30* in response to salt or drought stress.

## 3. Discussion

Drought and salt stresses are always discussed together because drought and salt have overlapping signals and complex secondary effects, including oxidative stress, damage to cellular components, and metabolic dysfunction. However, it is also necessary to distinguish the primary stress signals from secondary signals caused by too little water or too much salt [19]. The primary signal caused by drought is hyperosmotic stress, while salt stress has both osmotic and ion-toxicity effects on cells. MYBs as positive regulators for plants in response to drought or salt stress have been reported in many plants. In soybean (*Glycine max* L.), GmMYB84 overexpression enhanced drought resistance with higher antioxidant enzyme activity [peroxidase (POD), catalase (CAT), and superoxide dismutase (SOD)] and reduced malondialdehyde (MDA) content [20]. TaMYB30-B, a wheat R2R3-MYB gene, has been reported to improve drought stress tolerance in transgenic *Arabidopsis* [21]. In apple, MdMYB46 enhances salt and osmotic stress tolerance by activating secondary cell wall biosynthesis pathways and stress-responsive signals [22]. In addition, MYB transcription factors always show similar effects on plant tolerance to drought and salt stress. TaMYB33 overexpression increases both salt and drought tolerance in *Arabidopsis* [23]. A similar function in enhancing tolerance to drought and salt stress has also been characterized for grape VhMYB2, rice OsMYB6 and OsMYB48-1, and wheat TaODORANT1 [24,25,26,27]. In addition to these R2R3 MYB genes, the overexpression of OsMYB3R-2, an R1R2R3 MYB gene, increases tolerance to drought and salt stress in transgenic *Arabidopsis* [28]. In contrast, some MYBs, such as buckwheat FtMYB22, negatively regulate salt and drought stress through the ABA-dependent pathway [29]. Mulberry is considered as an economic tree with economic value, and some mulberry cultivars are promising pioneer trees for use on marginal land and in drought areas [30]. Zhongshen1 and Wubu are two mulberry varieties with quite different drought tolerance [11,16]. Under drought stress, Zhongshen1 showed decreased productivity and increased proline, abscisic acid, ROS content, and activity of antioxidant enzymes, while Wubu sustained comparable productivity and photosynthesis [16]. In this study, we first characterized *MaMYBR30*, a SHAQKY-class MYB (1R-MYB) in mulberry, and revealed its roles in regulating plant tolerance to drought and salt stress. MaMYBR30 showed quite different expression levels in Zhongshen 1 and Wubu under drought stress. Both the VIGS down-regulation of *MaMYBR30* in mulberry and the overexpression of *MaMYBR30* in transgenic *Arabidopsis* showed that *MYBR30* positively regulated drought stress tolerance while negatively regulating resistance to salt stress and affecting plant growth and flowering. Given the overlapping and unique effects of drought and salt stress on cells, it is likely that *MaMYBR30* plays quite different roles in regulating the signal pathway and genes involved in osmotic stress and ion toxicity. *MaMYBR30* overexpression in *Arabidopsis* resulted in more sensitivity to primary effects caused by salt stress. Co-expression analysis showed that calcium binding-related genes, including CDPK2, clustered in significantly negatively related modules (MEyellow or Mered), indicating that *MaMYBR30* overexpression inhibited the calcium signal pathway, which is important for alleviating ion toxicity. In turn, *MaMYBR30* showed positive co-expression with genes involved in response to water deprivation and oxidative stress, which helps to enhance drought stress tolerance.

Previous studies on MYBs mainly focus on R2R3-MYB, and studies on 1R-MYB are relatively insufficient. Reported 1R-MYBs mainly function as regulators involved in secondary metabolism, morphogenesis, and development [1,6]. In this study, we also found that *MaMYBR30* affects flower development and the transition of vegetable growth to reproductive growth, especially after exposure to salt stress. *MaMYBR30* is co-expressed with many genes involved in vernalization, flowering, and shoot system development. In addition, *MaMYBR30* also participates in regulating genes such as ATX1, ATX7, ASHH2, and SDG31, which are involved in epigenetic modification, including that of H3K4Me3, H3K27Me3, and H3K9Me3 (Table 1). These epigenetic modifications are involved in activating genes involved in flower development, such as FLC. The effects on flower development appear to become severe when exposed to salt stress instead of drought stress and result in abnormal flower and seed development. Therefore, these results imply that complex molecular mechanisms mediated by *MaMYBR30*, including crosstalk of ion toxicity, phytohormone signal transduction, flowering development, and epigenetic modification, need to be further explored in the future.

## 4. Materials and Methods

### 4.1. Plant Materials

The materials used in this study were obtained from the National Germplasm Resource Nursery of the Institute of Sericulture, Chinese Academy of Agricultural Sciences in Zhenjiang (north latitude 32°11′, east 119°27′), Jiangsu Province, China. Drought treatments of mulberry drought-sensitive cultivar Zhongshen 1 and drought-tolerant cultivar Wubu and their sample collection used for RNA sequencing (RNA-seq) to screen genes involved in drought stress response were reported in a previous study [16]. Leaves, buds, stems, and roots of one-year-old the *M. alba* variety *Fengchi* were collected for molecular cloning and tissue expression profile analysis. Drought, water-logging, low-temperature (4 °C), and high-temperature (40 °C) treatments were performed as previously reported and the leaves were collected for expression level measurement [31,32]. High-salt treatment was performed using different concentrations (100~300 mM) of NaCl irrigation until the observation of wilted, dark leaves and yellowing margins. Mulberry leaves were collected to determine *MYBR30* expression level changes in response to diverse stressors. *M. alba* var. *Fengchi* seedlings at the four-euphylla stage were used for virus-induced gene silencing (VIGS), as we reported in previous studies [33]. VIGS-treated seedlings with different *MaMYBR30* expression levels were collected for RNA-seq to identify co-expressed genes. Mulberry VIGS-treated seedlings showing the significant down-regulation of *MaMYBR30* compared with the controls were further treated under drought or salt stresses. *M. alba* var. *Fengchi seedlings* were transplanted into pots after gemination in moist dishes and were grown in a growth chamber at 22 °C with a 16/8 day/night cycle and 40–60% humidity. *Arabidopsis thaliana* (Ler) was used for transgenic experiments to obtain *MYBR30*-overexpressing *Arabidopsis* lines. Samples were immediately frozen in liquid nitrogen after collection and stored at −80 °C until use. Two or three biological replications were performed for each experiment.

### 4.2. RNA-Seq and Comparative RNA-Seq Analysis

The trimmed and filtered reads were aligned to the *M. alba* genome released by Jiao et al. (2020) using bowtie2 (version-2.3.2) [34]. Samtools was used to operate the bam files. StringTie v2.15 was used to calculate the expression matrix with the genome annotation file (.gff3) [35]. Tbtools 2.089 was used to obtain the differential expression genes [36]. Weighted correlation network analysis (WGCNA) was performed to screen the co-expressed DEGs [37]. For the RNA-seq dataset of drought-treated Zhongshen1 and Wubu, WGCNA was performed using water content and cultivars as associated traits. For the RNA-seq dataset of differentially expressed *MaMYBR30*, the expression level of *MaMYBR30* was used as the associated trait. R version 4.1.2 was used for R-package based analyses. Cytoscape 3.7.2 was used to visualize the co-expression network.

### 4.3. Cloning and Expression Profile Analysis of MaMYBR30 in Mulberry

Isolation of total RNA and cDNA synthesis were performed according to our previous reports. Primers used for cloning *MaMYBR30* were designed according to the sequence from *M. alba* with geneID M.alba_G0013567 (Table S2). The expression level of *MaMYBR30* in various organs and under various stresses were investigated by qRT-PCR (quantitative real-time PCR) using the ABI StepOnePlus™ Real-Time PCR System (Thermo Fisher Scientific, Foster, CA, USA). Then, 2× ChamQ™ SYBR^®^ qPCR Master Mix (Vazyme, Nanjing, China) with 50 × ROX Reference Dye 1 was used to prepare the reaction mix. The program used for the qRT-PCR was the two-step cycling protocol (95 °C for 2 min followed by 40 cycles of 95 °C for 10 s and 60 °C for 30 s) with Actin as the reference gene. Graphpad Prism8.0 was used to visualize the qRT-PCR results and ANOVA; *p* < 0.05 was marked as significant. Three biological replications or three technical replications were performed. 

### 4.4. Alignment and Phylogenetic Analysis of MaMYBR30

Alignment of *MaMYBR30* with other 1R-MYBs from *Arabidopsis thaliana* (KANADI-class AtMYBs and SHAQKYF-class MYBs) was performed using DNAman 8.0 (Lynnon BioSoft, San Ramon, CA, USA) with default parameters. The MYB DNA-binding motif and SHAQKYF motif were scanned and marked. In addition, both 1R-MYBs from mulberry [15] and *Arabidopsis thaliana* [7] were collected and aligned to construct a maximum-likelihood phylogenetic tree using Mega X with the JTT substitution model, the G+I rates among sites model, and 1000 bootstrap replicates [38,39]. 

### 4.5. Subcellular Location of MaMYBR30

The method used for the subcellular localization of *MaMYBR30* was according to our previous study [40]. The confirmed recombinant plasmids constructed using Nimble cloning [41] were transferred into *Agrobacterium tumefaciens* strain GV3101, which was then transferred into tobacco leaves via Agrobacterium-mediated transient transformation together with recombinant plasmid pBin-Sv40-NLS-mCherry as the nuclear localization marker [42]. YFP and mCherry fluorescence signals in leaves were observed with a Leica TCS SP8 confocal microscope (Leica Microsystems, Wetzlar, Germany).

### 4.6. VIGS Treatment of Mulberry

We generated mulberry trees with different degrees of down-regulated expression levels of *MaMYBR30* using virus-induced gene silencing (VIGS) [13]. Nimble cloning was used to construct the recombinant plasmids used for VIGS [41]. VIGS in mulberry was performed according to our previous studies [13,32,33]. Pressure injection was adopted to infect the mulberry leaves, and empty vectors pTRV2 and pTRV1 were used as negative controls. The expression level of *MaMYBR30* was detected using qRT-PCR, 15 days after injection. The knock-down efficiency of *MaMYBR30* was calculated by comparing the expression level of *MaMYBR30* in VIGS-treated plants with the controls. 

### 4.7. Overexpression of MaMYBR30 in Arabidopsis

Recombinant plasmids pCambia1304-35S::MaMYB30 were constructed using Nimble cloning and confirmed by sequencing. The recombinant plasmids were then transformed into *Agrobacterium tumefaciens* strain GV3101, as described above. The floral dip method was adopted to obtain the transgenic *Arabidopsis* seeds, and further, positive seedlings were screened using Hygromycin B (20 mg/L). Genomic DNA was extracted as a template and PCR was performed to further determine the fusion of *MaMYBR30* into the *Arabidopsis* genome. About nine individual transgenic lines were generated, and four lines were selected for further analysis. The T3 progenies of selected transgenic lines were germinated on agar plates containing half of Murashige and Skoog medium (1/2MS), and the transgenic seedlings were further confirmed by qRT-PCR to detect the overexpression of *MaMYBR30* in *Arabidopsis* compared to the WT (Ler). These *MaMYBR30*-overexpression transgenic *Arabidopsis* plants were used for observation and drought and salt treatments.

### 4.8. Drought and Salt Stress Treatment and Physiology Indicator Determination

Mulberry plants with validated down-regulated *MaMYBR30* by VIGS and progenies of selected transgenic *Arabidopsis* lines were exposed to drought or salt stresses. Plants exposed to drought stress were placed in one pot, and the tests were carried out by stopping irrigation, and the controls were irrigated with water normally. Plants exposed to high salt stresses were treated every day with 100, 150, 200, and 300 mM NaCl instead of water. Both treatments were kept for eight days, and then, the plants were recovered by water irrigation. MDA contents and SOD activity in leaves were determined using the samples collected on the eighth day, as we described in a previous study [43]. Briefly, the MDA content was determined as described by Sairam and Srivastava (2001) [44]. SOD activity was determined using a SOD measurement kit (Suzhou Keming Technology, Suzhou, China) according to the manufacturer. Graphpad Prism8.0 was used to perform ANOVA and visualize the results. *p* < 0.05 was considered significant. All above measurements were carried out with three biological replications. Three newly grown leaves of two independent VIGS-treated mulberry plants were used as biological replicates.

## 5. Conclusions

In conclusion, we report a 1R SHAQKYF-class MYB, *MaMYBR30*, and its function in mulberry. *MaMYBR30* is localized in the nucleus and belongs to the CCA-like subgroup with a conserved SHAQKYF motif. The functional characterization of *MaMYBR30* in vivo indicated that *MaMYBR30* can positively regulate the resistance of mulberry to drought while negatively regulating the resistance of mulberry to salt stress. In addition, *MaMYBR30* also affects flower development and reproductive growth, especially after exposure to salt stress. Complex molecular mechanisms mediated by *MaMYBR30* need to be further explored in the future. 

## Figures and Tables

**Table 1:** Annotation of DEGs from co-expression network of *MaMYBR30*.

*Morus* Gene ID	Homolog in *Arabidopsis*	Symbol	Bioprocess	Modules
M.alba_G0001459	AT1G35670	ATCDPK2	calmodulin binding	yellow
M.alba_G0004615	AT4G35580	NTL9	calmodulin binding	red
M.alba_G0010384	AT2G19130	AT2G19130	calmodulin binding	red
M.alba_G0000522	AT5G48670	AGL80	seed development	red
M.alba_G0005385	AT1G13290	DOT5	leaf development	red
M.alba_G0003162	AT5G06720	ATPA2	flower development, oxidative stress	yellow
M.alba_G0008384	AT3G04380	SDG31	H3K9Me3	yellow
M.alba_G0001138	AT1G77300	ASHH2	H3K36Me, flower	turquoise
M.alba_G0004262	AT2G31650	ATX1	H3K4Me3, flower	turquoise
M.alba_G0004291	AT5G42400	ATXR7	H3K4Me3, flower	turquoise
M.alba_G0001922	AT3G23230	AtERF98	tolerance to salt	turquoise
M.alba_G0002850	AT1G14350	AtMYB124	leaf development	turquoise
M.alba_G0009116	AT3G24440	VIL1	flower development	turquoise
M.alba_G0002773	AT3G06110	ATMKP2	MAPK cascade	turquoise
M.alba_G0002771	AT4G29810	ATMKK2	MAPK cascade	turquoise
M.alba_G0007116	AT3G18040	MPK9	MAPK cascade	turquoise
M.alba_G0005503	AT5G55390	EDM2	regulation of flower development	turquoise
M.alba_G0004249	AT4G24240	ATWRKY7	calmodulin binding	turquoise
M.alba_G0006983	AT3G02570	MEE31	ABA-activated signaling pathway	turquoise
M.alba_G0001350	AT2G41070	ATBZIP12	ABA-activated signaling pathway	turquoise
M.alba_G0000876	AT1G07570	APK1	response to oxidative stress	turquoise
M.alba_G0002641	AT1G66730	AtLIG6	response to oxidative stress	turquoise
M.alba_G0000024	AT1G51680	4CL.1	lignin biosynthesis	turquoise
M.alba_G0004912	AT1G09340	CRB	response to water deprivation	turquoise
M.alba_G0002629	AT4G02380	AtLEA5	response to water deprivation	turquoise
M.alba_G0002247	AT1G78380	ATGSTU19	response to water deprivation	turquoise
M.alba_G0001324	AT3G06190	ATBPM2	response to water deprivation	turquoise
M.alba_G0001327	AT3G03740	ATBPM4	response to water deprivation	turquoise

## Data Availability

The original contributions presented in the study are included in the article/Supplementary Materials, further inquiries can be directed to the corresponding author.

## Supplementary Materials

The following supporting information can be downloaded at https://www.mdpi.com/article/10.3390/plants13131794/s1: Table S1: Expression matrix of RNA-seq dataset involved in drought stress responsiveness; Table S2: Primers used in this study; Table S3: DEGs caused by change in MaMYBR30 expression level; Figure S1: Venn diagram of drought-induced DEGs and abiotic stress-related MYBs; Figure S2: Phylogenetic analysis of 1R-MYB from *Morus alba* and *Arabidopsis thaliana*; Figure S3: Growth condition of transgenic and wild-type *Arabidopsis*; A. Growth condition of transgenic and wild-type Arabidopsis with normal water supply; B. qRT-PCR detection of MaMYBR30 expression levels in transgenic Arabidopsis lines; C. Growth condition of transgenic and wild-type Arabidopsis under salt stress by irrigation with 100 mM, 200 Mm, and 300 mM NaCl instead of water; D. Reproductive growth of transgenic and wild-type Arabidopsis after exposure to salt stress and recovery with water supply. CK: wild type (Ler); MYBR30#1, 2, 3: MaMYBR30-overexpressing lines. Figure S4: qRT-PCR detection of MaMYBR30 expression levels of different sample groups; Figure S5: GO enrichment analysis of top 300 MYBR30-related genes.
