# Supplementary figures and images for: MaMYBR30, a Novel 1R-MYB, Plays Important Roles in Plant Development and Abiotic Stress Resistance
Source: Plants (Basel). 2024 Jun 28;13(13):1794. doi: 10.3390/plants13131794 (PMC11244220; doi:10.3390/plants13131794)

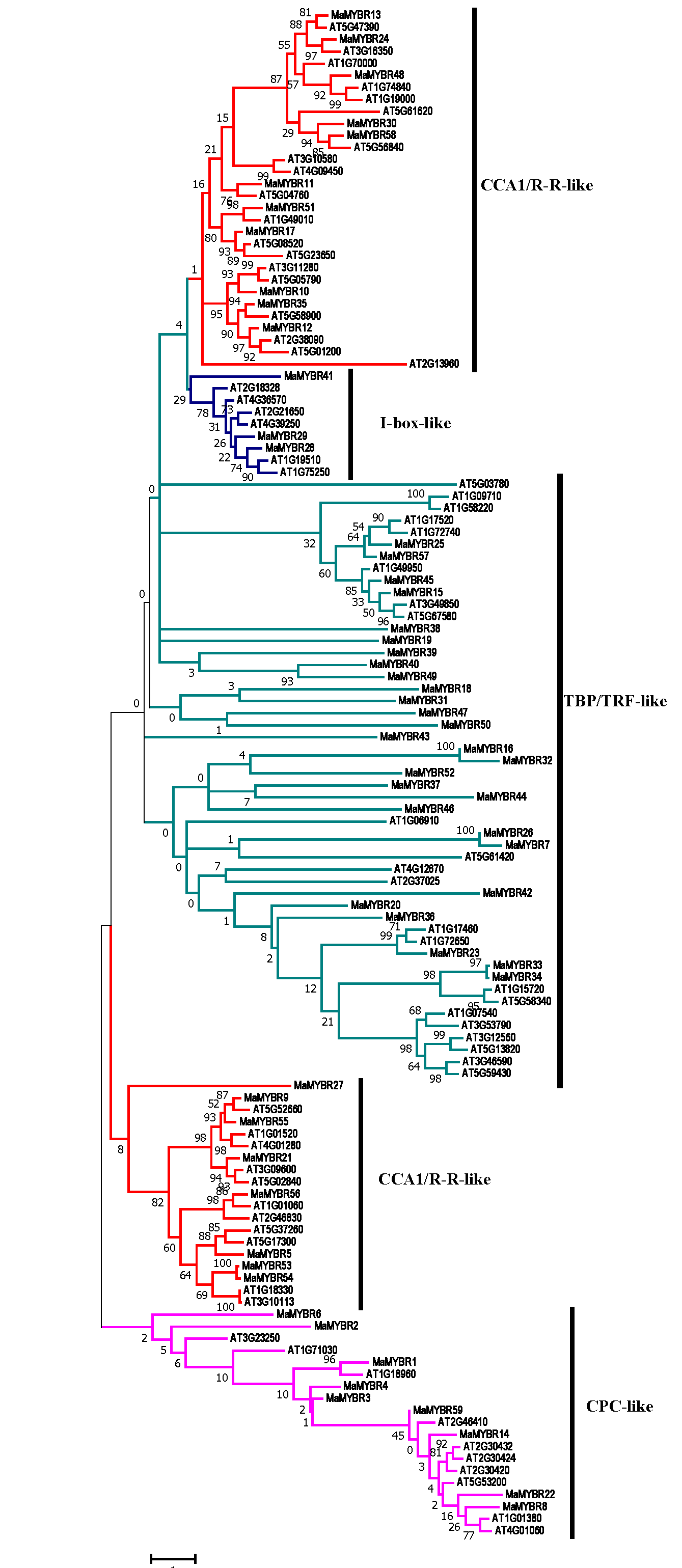

Supplement: Supplementary file 1 [file plants-13-01794-s001.zip › Figure S2 cpc-like-3-21.tif]

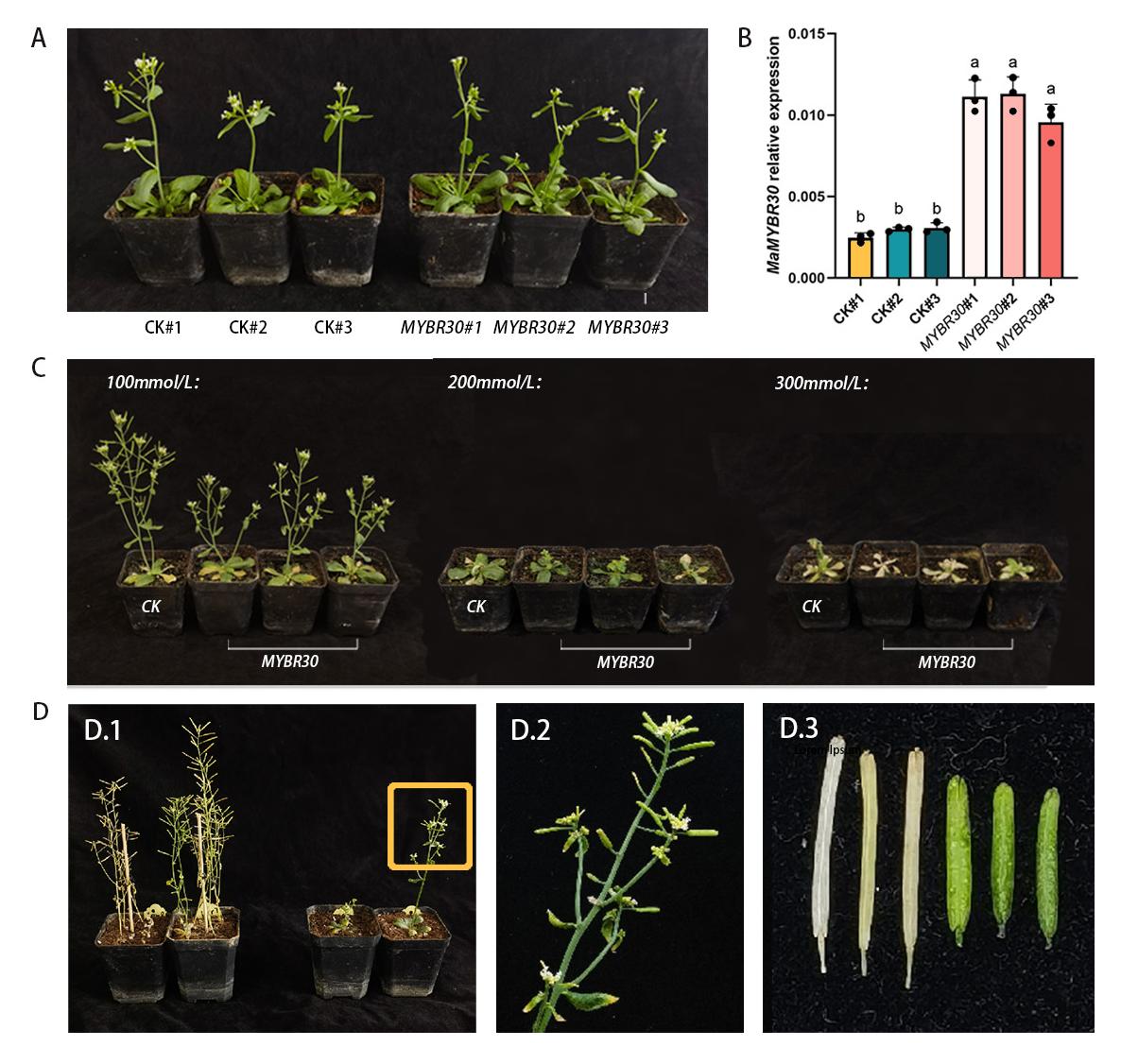

Supplement: Supplementary file 1 [file plants-13-01794-s001.zip › Figure S3.jpg]

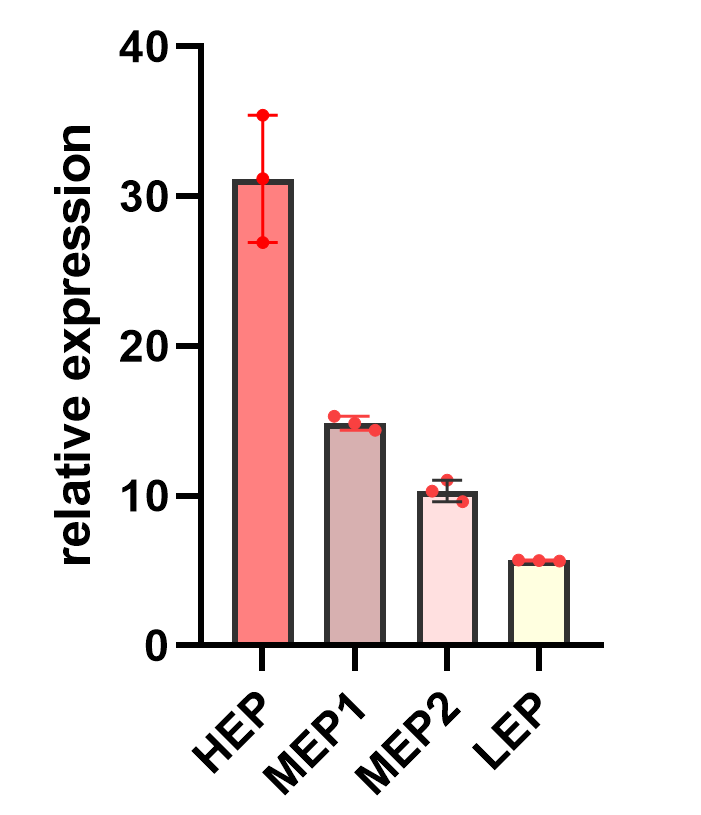

Supplement: Supplementary file 1 [file plants-13-01794-s001.zip › Figure S4.tif]

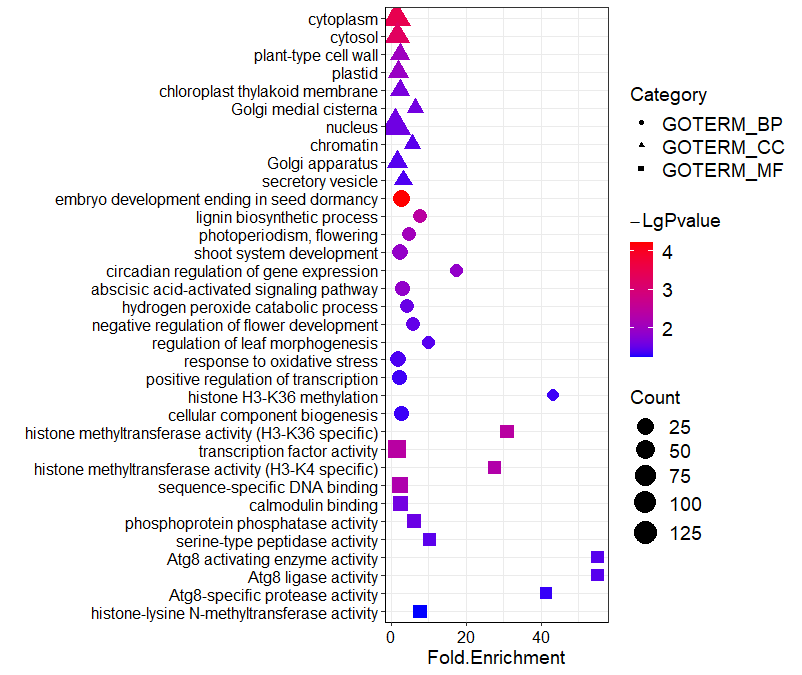

Supplement: Supplementary file 1 [file plants-13-01794-s001.zip › Figure S5-MYBR30-TOP300.tiff]

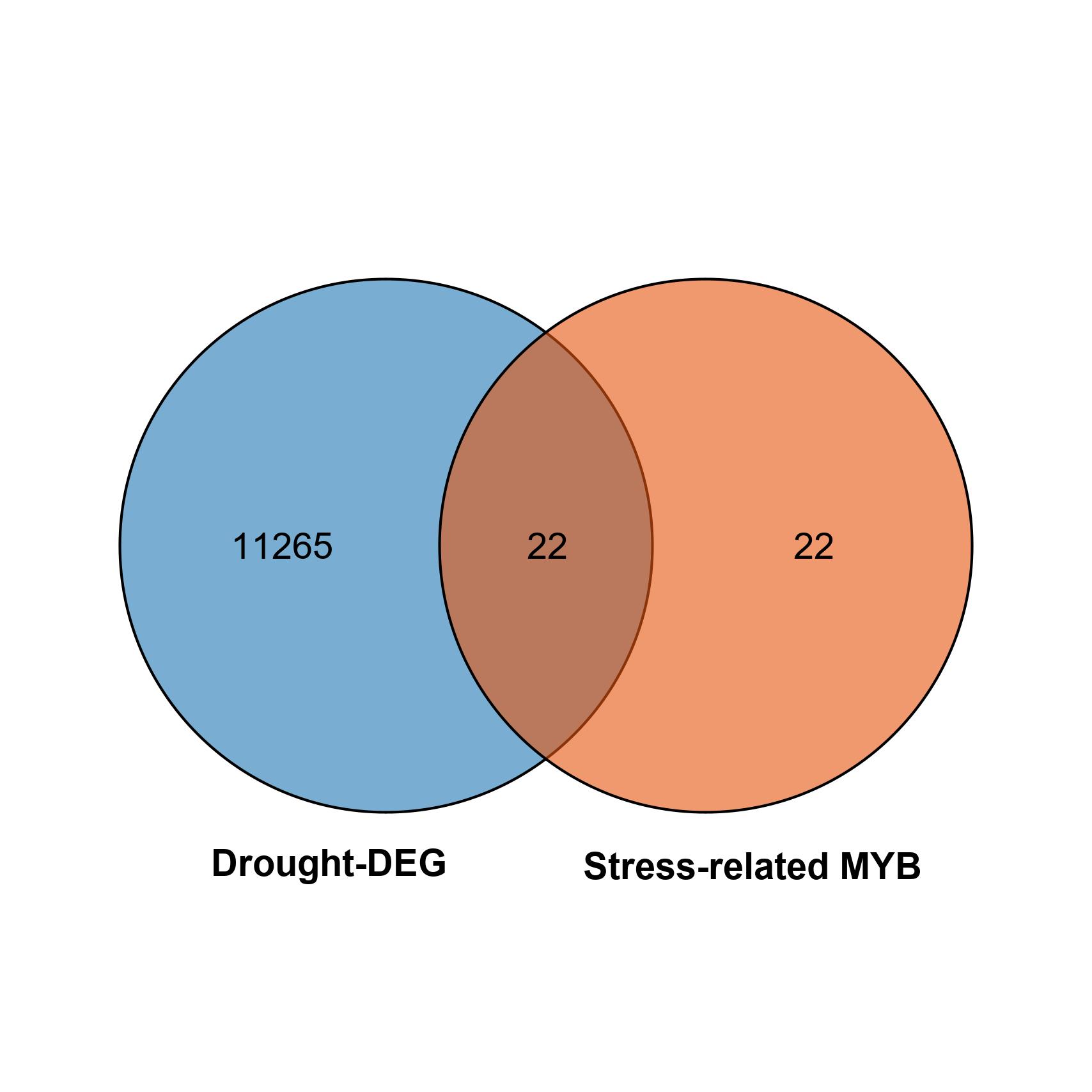

Supplement: Supplementary file 1 [file plants-13-01794-s001.zip › FigureS1.jpg]
